# Protective Effect of Ginsenoside Rb1 Against Lung Injury Induced by Intestinal Ischemia-Reperfusion in Rats

**DOI:** 10.3390/molecules18011214

**Published:** 2013-01-17

**Authors:** Jin Wang, Lifen Qiao, Shusheng Li, Guangtian Yang

**Affiliations:** 1 Department of Emergency Medicine, Tongji Hospital, Tongji Medical College, Huazhong University of Science and Technology, 1095 Jiefang Avenue, Wuhan 430030, Hubei, China; 2 General Respiratory Department, Tongji Hospital, Tongji Medical College, Huazhong University of Science and Technology, 1095 Jiefang Avenue, Wuhan 430030, Hubei, China

**Keywords:** ischemia reperfusion, tumor necrosis factor alpha, intracellular adhesion molecule-1, nuclear factor kappa B, ginsenoside Rb1

## Abstract

Intestinal ischemia-reperfusion (I/R) is a critical event in the pathogenesis of multiple organ dysfunction syndromes (MODS). The lungs are some of the most vulnerable organs that are impacted by intestinal I/R. The aim of this study is to investigate whether ginsenoside Rb1 can ameliorate remote lung injury induced by intestinal I/R. Adult male Wistar rats were randomly divided into four groups: (1) a control, sham-operated group (sham group); (2) an intestinal I/R group subjected to 1 h intestinal ischemia and 2 h reperfusion (I/R group); (3) a group treated with 20 mg/kg ginsenoside Rb1 before reperfusion (Rb1-20 group); and (4) a group treated with 40 mg/kg ginsenoside Rb1 before reperfusion (Rb1-40 group). Intestinal and lung histology was observed. The malondialdehyde (MDA) levels in intestinal tissues were measured. Myeloperoxidase (MPO), TNF-α, MDA levels, wet/dry weight ratio and immunohistochemical expression of intracellular adhesion molecule-1 (ICAM-1) in lung tissues were assayed. In addition, a western blot of lung NF-kB was performed. Results indicated that intestinal I/R induced intestinal and lung injury, which was characterized by increase of MDA levels and pathological scores in intestinal tissues and MPO, TNF-α , MDA levels, wet/dry weight ratio and ICAM-1, NF-kB expression in the lung tissues. Ginsenoside Rb1 (20, 40 mg/kg) ameliorated intestinal and lung injury, decreased MPO, TNF-α, MDA levels, wet/dry weight ratio, ICAM-1 and NF-kB expression in lung tissues. In conclusion, ginsenoside Rb1 ameliorated the lung injuries by decreasing the NF-kB activation-induced inflammatory response.

## Abbreviations

I/R: ischemia-reperfusion
MODS: multiple organ dysfunction syndrome
MDA: malondialdehyde
MPO: myeloperoxidase
TNF-α: tumor necrosis factor α
ICAM-1: intracellular adhesion molecule -1
NF- kB: nuclear factor kappa B
Rb1: ginsenoside Rb1

## 1. Introduction

Intestinal ischemia reperfusion (I/R) is common in critically ill patients, which can result in not only intestinal injury, but also injury in distant organs. The lungs are some of the most vulnerable organs that are impacted by intestinal I/R. Local inflammatory changes play a pivotal role in the development of acute lung injury after intestinal I/R, but the underlying molecular mechanisms are not fully understood. 

Studies demonstrate that during intestinal I/R, neutrophils, cytokines such as tumor necrosis factor-alpha (TNF-α), free radicals, and chemokines are included in the wide spectrum of inflammatory mediators involved in the physiopathology of intestinal I/R-induced tissue injury [1]. Moreover, leukocyte-endothelial interaction plays a critical role in lung injury, recruiting and activating neutrophils to release inflammatory mediators. In this context, ICAM-1 exerts pivotal control over the movement of neutrophils into the injured site [2]. Recent studies have shown that these mediators are regulated by a nuclear factor kappa B (NF-kB) family of transcription factors, which is a key regulator of inflammatory gene expression. And NF-kB is increasingly implicated in the development of lung injury during intestinal I/R [3]. 

Ginsenosides, which are glycosides containing an aglycone (protopanaxadiol or protopanaxatriol) with a dammarane skeleton, are the major effective components of ginseng and have been shown to have a wide variety of biological activities including immunomodulatory effects, antioxidant, anti-inflammatory and anti-tumor activity [4,5,6,7].

**Figure 1:**
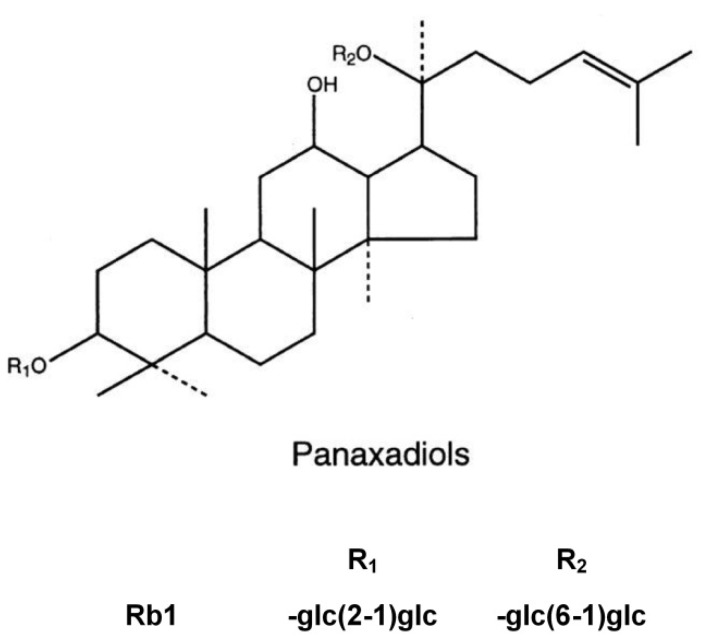
The molecular structure of Rb1.

Ginsenosides are normally fractioned into two groups based on the types of aglycone, namely the panaxadiol group (e.g., Rb1 and Rc, Figure 1) and the panaxatriol group (e.g., Rg1 and Re). Ginsenoside Rb1, one of the panaxadiols, showed anti-stress effects in acute, chronic, and repeated stress models [8]. It has beneficial effects on cerebral [9], myocardial [10] ischemia reperfusion injury and intestinal I/R induced renal injury [11]. Our previously research had also suggested that ginsenoside Rb1 can attenuate intestinal I/R induced liver injury by inhibiting NF-kB activation [12], but the effect of ginsenoside Rb1 on intestinal I/R induced lung injury has not been reported. In this paper, we investigate the influence of ginsenoside Rb1 on lung injury and NF-kB activation induced by intestinal I/R.

## 2. Results and Discussion

### 2.1. Effects of Rb1 on Intestinal Injury Induced by Intestinal I/R

**Figure 2:**
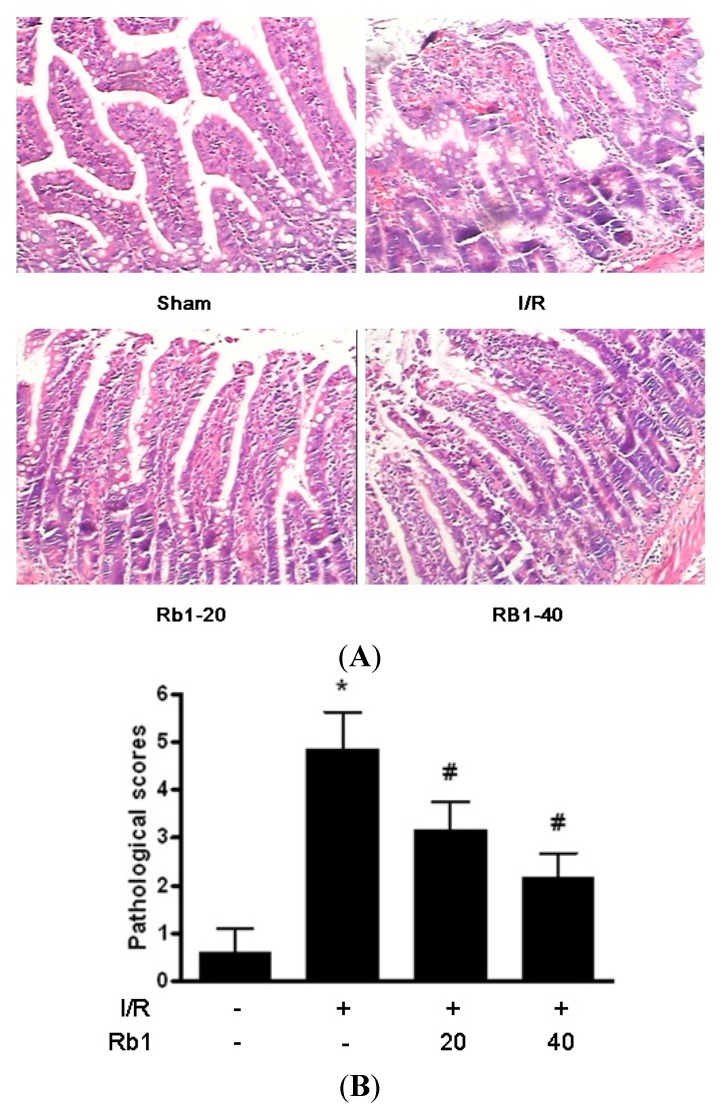
(**A**) Effects of Rb1 on intestinal injury after intestinal I/R. (HE ×400); (**B**) Intestinal pathological scores of different groups.

Occluded small intestine showed a dark color and mild swelling after 1 h intestinal ischemia and 2 h reperfusion. The edema, bleeding and villi irregularities could be found in the intestinal mucosa and submucosa in the I/R group. Treatment of rats with Rb1 resulted in a marked amelioration of intestinal injury compared with the I/R group. Pathological scores are expressed as means ± S.D. * *p* < 0.01 *versus* sham group; ^#^ *p* < 0.01 *versus* I/R group (Figure 2).

### 2.2. Effects of Rb1 on Lung Injury Induced by Intestinal I/R

**Figure 3:**
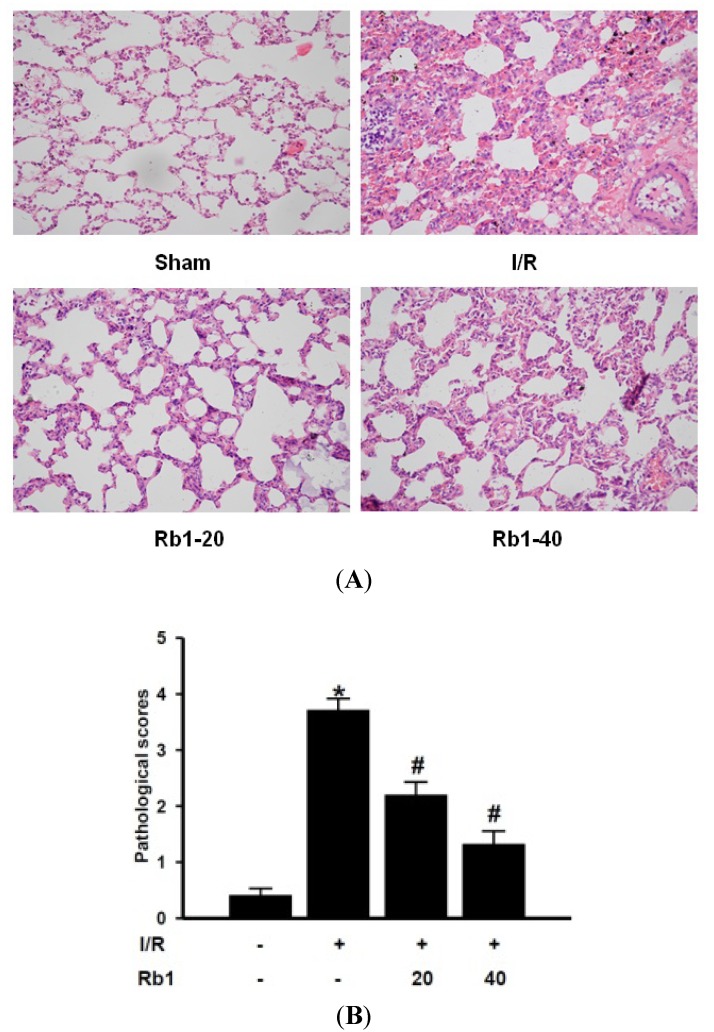
(**A**) Effects of Rb1 on lung injury after intestinal I/R. (HE ×400); (**B**) Lung pathological scores of different groups.

Lung injury manifested as the histological characteristics of lung edema, hemorrhage and PMN infiltration. The sham group showed normal appearance of lung tissue. The I/R group showed the majority of the interstitial capillaries congested with erythrocytes, with hemorrhage and edema between alveoli. A great number of inflammatory cells infiltration increased septal thickness. Significant amelioration of histological oedema, haemorrhage and inflammatory cell infiltration was seen in the 20 and 40 mg/kg Rb1 treated groups. Pathological scores are expressed as means ± S.D. * *p* < 0.01 *versus* sham group; ^#^ *p* < 0.01 *versus* I/R group (Figure 3). The mechanism underlying the attenuation of intestinal I/R induced lung injury by Rb1 is the emphasis of this study.

### 2.3. Effects of Rb1 on MDA Levels in Intestinal and Lung Tissues

Oxidative stress is one proposed mechanism for the development of I/R-induced damage. Lipidic peroxidation is frequently used to prove the involvement of free radicals in cell damage. MDA is one of the final products of lipidic peroxidation and several methods have been proposed for its determination. MDA levels are directly proportional to the cell damage caused by free radicals [13]. 

Following 1 h of intestinal ischemia, reperfusion significantly increased intestinal tissue MDA levels in the I/R group compared with sham group. Administration of 20 mg/kg and 40 mg/kg Rb1 markedly decreased intestinal MDA levels compared with the I/R group. Data represent means ± S.D. * *p* < 0.01 *versus* sham group;^ #^ *p* < 0.01 *versus* I/R group.

We also examined the effects of Rb1 on lung tissue MDA levels. The lung tissue MDA levels were increased significantly in I/R group compared with the sham group. Administration of 20 mg/kg and 40 mg/kg Rb1 significantly reduced MDA levels. Data represent means ± S.D. * *p* < 0.01 *versus* sham group; ^#^ *p* < 0.01 *versus* I/R group.

**Figure 4:**
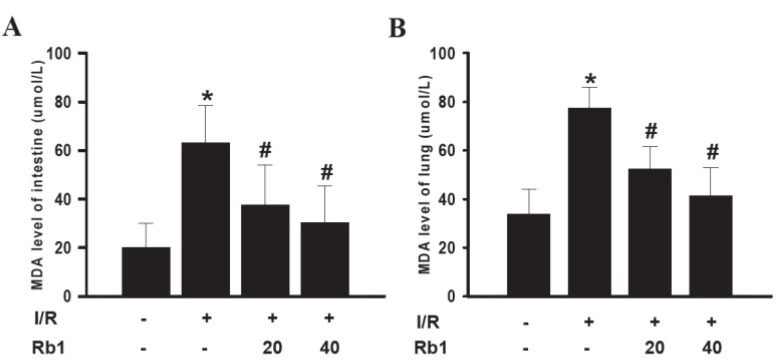
Effects of Rb1 on MDA levels of intestinal and lung tissues.

Results indicated that there existed free radical damage in lung and intestinal tissue and Rb1 could attenuate lung and intestinal damage caused by free radical (Figure 4).

### 2.4. Effects of Rb1 on Lung Wet/Dry Ratio Induced by Intestinal I/R

**Figure 5:**
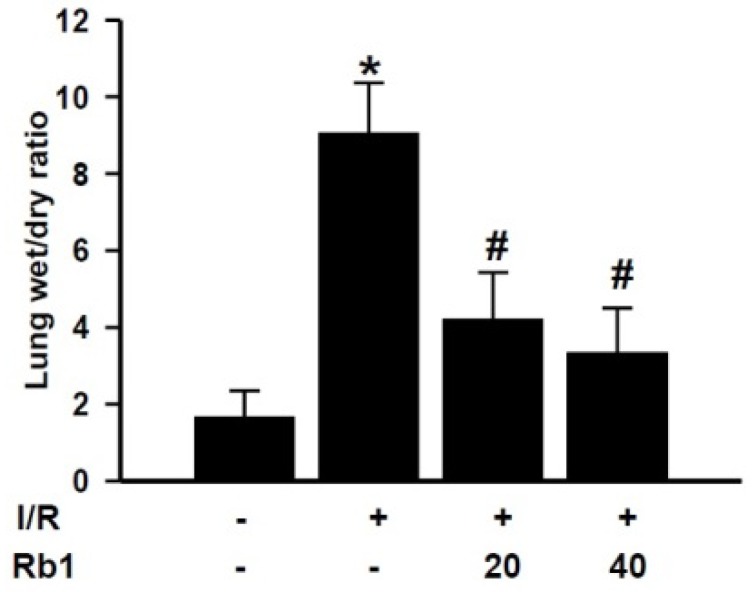
Effects of Rb1 on lung wet weight to dry weight ratio.

After 2 h reperfusion, lung wet/dry ratio was increased compared with the sham group, and Rb1 decreased the lung wet/dry ratio. Data represent means ± S.D. * *p* < 0.01 *versus* sham group; ^#^ *p* < 0.01 *versus* I/R group (Figure 5).

### 2.5. Effects of Rb1 on TNF-α Levels in Lung Tissue

**Figure 6:**
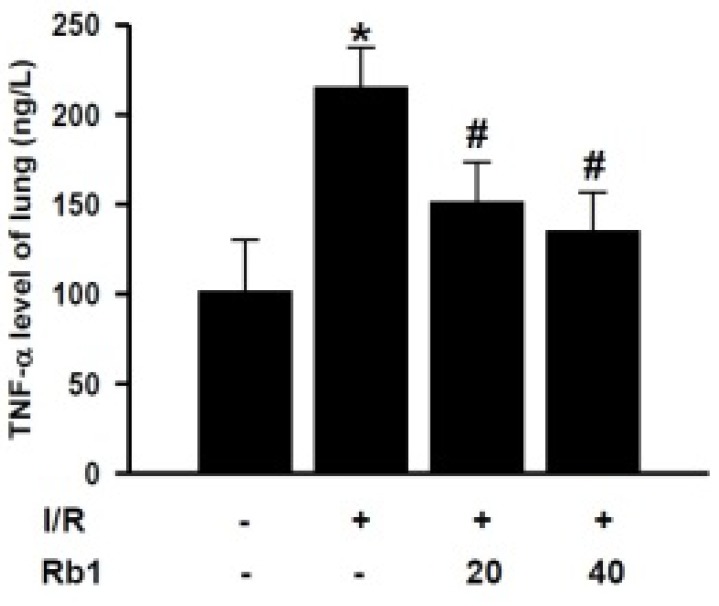
Effects of Rb1 on TNF-α levels of lung.

In order to investigate the mechanism of Rb1 protective role on lung injury, we studied the effects of Rb1 on the TNF-α levels in lung tissue. Compared with the sham group, lung tissue TNF-α levels increased significantly in the I/R group. Administration of 20 mg/kg and 40 mg/kg Rb1 significantly reduced TNF-α levels compared with I/R group. Data represent means ± S.D. * *p* < 0.01 *versus* sham group; ^#^ *p* < 0.01 *versus* I/R group (Figure 6).

### 2.6. Effects of Rb1 on MPO Activity in Lung Tissues

**Figure 7:**
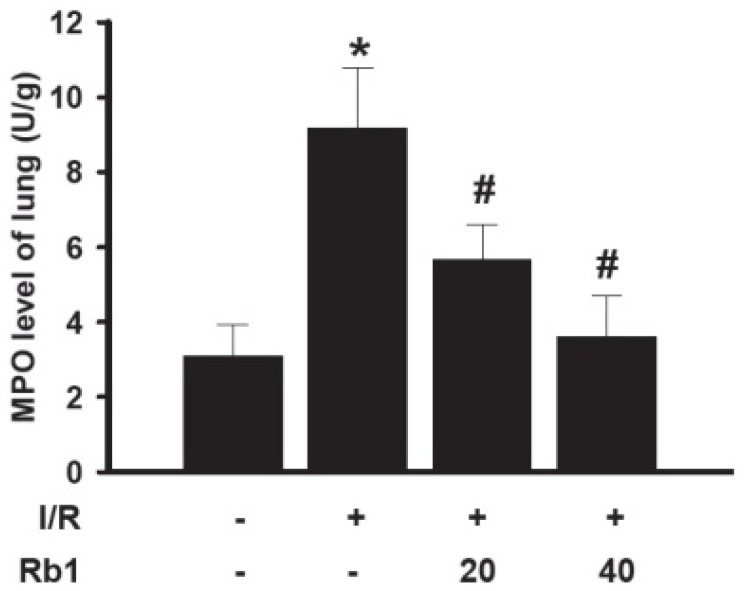
Effects of Rb1 on MPO levels of lung.

Neutrophil infiltration in lung tissue plays an important role in lung injury induced by intestinal I/R. The MPO assay is widely used to quantify the number of neutrophils in tissues and serves as an index of inflammation, because MPO is an enzyme that is released mainly from neutrophils. Compared with the sham group, the lung MPO activity was increased in I/R group. Treatment of rats with 20 mg/kg and 40 mg/kg of Rb1 significantly decreased MPO activity compared with the I/R group. Data represent means ± S.D. * *p* < 0.01 *versus* sham group; ^# ^*p* < 0.01 *versus* I/R group (Figure 7). There is a growing body of evidence that incriminates PMNs in the pathophysiological progress of I/R injury. The increased MPO activity in the lung tissue after intestinal I/R injury suggested activation of an inflammatory response.

### 2.7. Effects of Rb1 on Lung ICAM-1 Expression by Immunohistochemical Analysis

**Figure 8:**
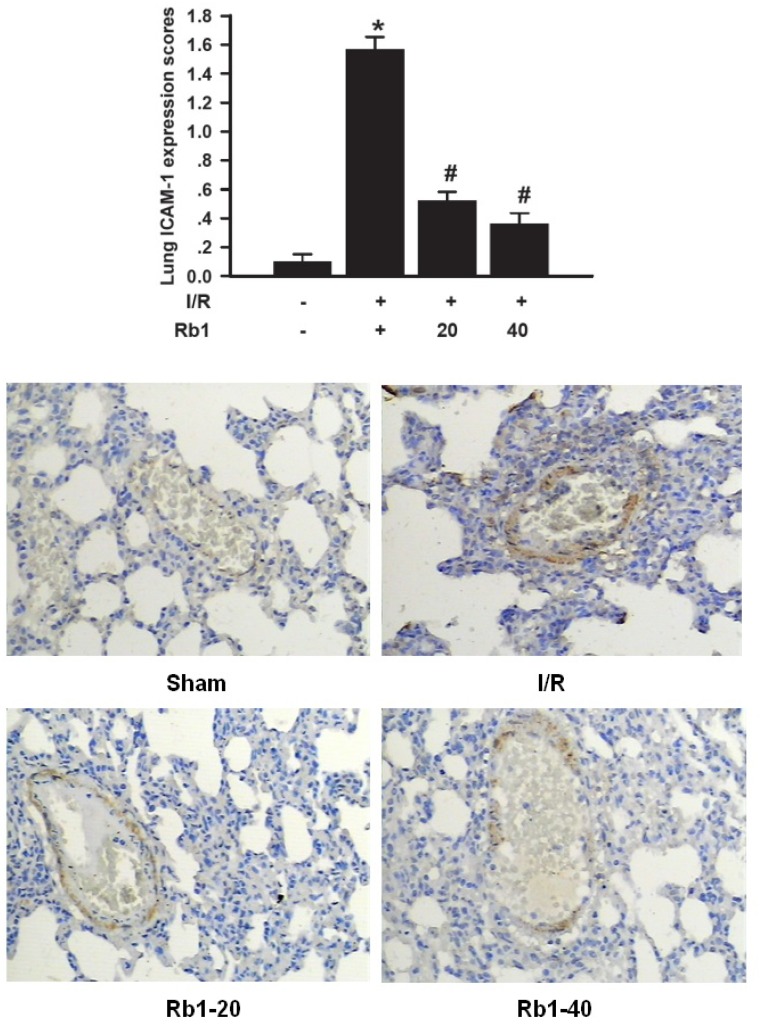
Effects of Rb1 on ICAM-1 expression of lung tissue after intestinal I/R (×200).

Histological examination and MPO assay in lung tissue showed more extensive neutrophil infiltration in the I/R group. We therefore studied the effects of Rb1 on expression of the ICAM-1 which is required for neutrophil recruitment in lung tissue. ICAM-1 expression immunohistochemical analysis showed little staining in the sham group. Significant positive expression of ICAM-1 with strong brown staining was observed in the I/R group. The expression of ICAM-1 was decreased in Rb1-20 and Rb1-40 group compared with the I/R group. Data represent means ± S.D. * *p* < 0.01 *versus* sham group; ^#^ *p* < 0.01 *versus* I/R group (Figure 8). ICAM-1 plays a key role in neutrophil chemoattraction, adhesion, and emigration from the vascularture to the tissue, contributing the systemic inflammatory response and organ injury [13]. Intestine-and/or lung-derived mediators, such as TNF-α, have been implicated as participants in the intestinal I/R-induced leucocyte mediated lung injury [14]. These data suggested that Rb1 attenuated lung injury by decreasing MPO activity, ICAM-1 expression and TNF-α levels in lung tissue.

### 2.8. Effects of Rb1 on Lung NF-kB by Western Blot Analysis

Western blot analysis showed weak NF-kB p65 positive signals in the lungs of the sham group. In contrast, significant increase of the NF-kB p65 protein expression was found in the I/R group. Compared with the I/R group, the signals were weakened in the Rb1-20 and Rb1-40 group. The data suggested that Rb1 markedly inhibited NF-kB activation with increasing dose of Rb1. These findings suggested the importance of NF-kB pathway in protective effects of Rb1 on lung injury induced by intestinal I/R. Data represent means ± S.D. * *p* < 0.01 *versus* sham group; ^#^ *p* < 0.01 *versus* I/R group.

Our previous work had shown that liver injury associated with intestinal I/R appears to be dependent on leucocyte adhesion and NF-kB activation [12]. Many studies have demonstrated that inflammatory mediators are regulated by NF-kB, which is a key regulator of inflammatory gene expression in lung injury. Modulating the NF-kB pathway is a new concept and therapeutic strategy to attenuate the lung injury caused by intestinal I/R.

**Figure 9:**
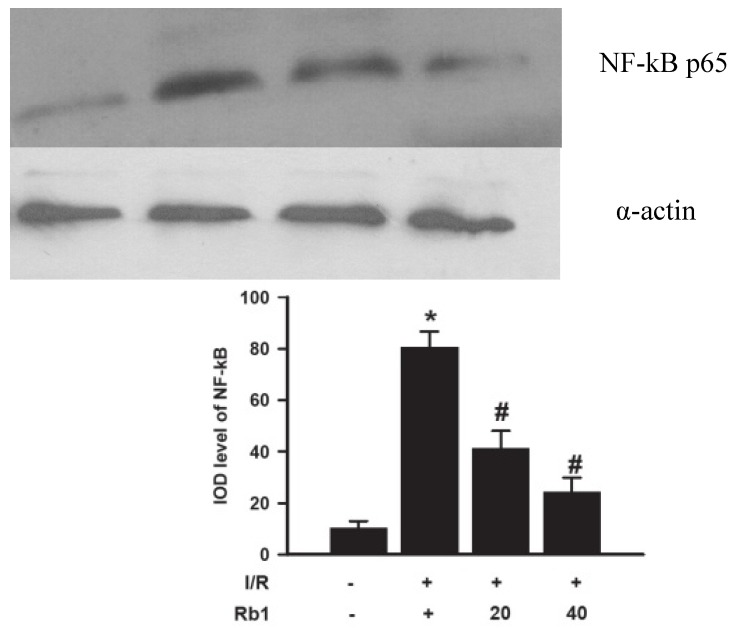
Effects of Rb1 on NF-kB activation of lung tissue after intestinal I/R.

NF-kB plays an important role in regulating activation of inflammatory mediators (ICAM-1, TNF-α) and the regulation of neutrophil aggregation [3]. Because NF-kB activation can lead to enhanced expression of proinflammatory cytokines, chemokines and adhesion molecules, modulation of NF-kB activation may provide a direct way of inhibiting inflammatory mediators [15]. For that reason, control of NF-kB activation is a potential therapeutic strategy to reduce the untoward tissue damage. Directing drug discovery efforts towards NF-kB activation rather than towards any one of its many target genes could produce a much greater therapeutic benefit by inhibiting expression of the constellation of NF-kB-induced pro-inflammatory genes [16]. In our study, 1 h intestinal ischemia followed by 2 h reperfusion induced lung injury that paralleled the increased levels of lung water content, MDA, MPO, TNF-αand ICAM-1 expression, as well as the expression of NF-kB. This suggests that NF-kB activation is involved in the pathogenesis of lung injury induced by intestinal I/R. And Rb1 could attenuate intestinal injury by decreasing intestinal free radicals damage and inhibiting the NF-kB activation induced inflammatory cytokines (MDA, MPO, TNF-α, ICAM-1) in the lung tissues in a dose-dependent manner. In the early stage of intestinal ischemia-reperfusion, the gut barrier function is progressively damaged, and bacteria, endogenous endotoxin, bacteriotoxin and reactive oxygen species (ROS) invade into the circulation, and the low levels of ROS and inflammatory mediators can activate the expression of NF-kB [17]. So the possible protective mechanism of inhibitory effects of Rb1 on NF-kB activation might be related to antioxidant and anti-inflammatory role of Rb1 (Figure 9).

## 3. Experimental

### 3.1. Materials

Adult male Wistar rats, weighing 220–250g, were obtained from Experimental Animal Center of Tongji Medical College (Grade II, and Certificate No 19-050). This study complies with the European Community Guidelines for the Care and Use of Experimental Animals and was approved by the Animal Research Committee of Tongji Medical College of Huazhong University of Science and Technology (Wuhan, China). Ginsenoside Rb1 (99.5%) were obtained from the Research Center of Traditional Chinese Medicine (Wuhan, China) and dissolved in saline. The MDA and MPO assay kits were obtained from Nanjing Jincheng Bioengineering Institute (Nanjing, China). TNF-α ELISA assay kit was obtained from R&D Systems, Inc (Minneapolis, MN, USA). Antibodies for NF-kB and α-actin were purchased from Santa Cruz Biotechnology, Inc (Santa Cruz, CA, USA). Nuclear Extract Kit was purchased from Active Motif, Inc (Carlsbad, CA, USA). Antibody for ICAM-1 was purchased from Boster Biological Technology, LTD (Wuhan, China). All other chemicals used were of the highest grade available commercially. The molecule structure of Rb1 is shown in Figure 1. 

### 3.2. Experimental Protocol

The animals were anesthetized with sodium pentobarbital (50 mg/kg) intraperitoneally. The intestinal I/R model is established by superior mesenteric artery (SMA) occlusion. Rats were randomly divided into four groups (n = 10 in each group): (1) sham-operated group (sham group) that underwent isolation of the superior mesenteric artery (SMA) without occlusion; (2) intestinal I/R group (II/R group) subjected to 1 h intestinal ischemia and 2 h reperfusion after the SMA had been isolated and occluded with administration of 10 mL/kg saline intraperitoneally before reperfusion [18]; (3) 20 mg/kg Rb1 treated group (Rb1-20 group), in which surgery was performed as in the intestinal I/R group with administration of 10 mL/kg 0.2% Rb1 (dissolved in saline) intraperitoneally before reperfusion; and (4) 40 mg/kg Rb1 treated group (Rb1-40 group), in which surgery was performed as in the I/R group with administration of 10mL/kg 0.4% Rb1 (dissolved in saline) intraperitoneally before reperfusion. Lung and intestinal tissues were obtained for analysis at the end of the 2 h reperfusion period.

### 3.3. Intestinal and Lung Histopathological Assessment

The isolated intestine tissues were cut into sections and fixed in 40 g/L formaldehyde. After being embedded in paraffin, 4 μm sections were stained with haematoxylin and eosin for light microscopy. Intestinal damage was evaluated by a pathologist who scored the histology using the system described by Chiu as follows: Grade 0, normal mucosa; Grade 1, subepithelial space developing at the tip of the villus; Grade 2, extension of the subepithelial space with moderate lifting of the epithelial layer from the lamina propria; Grade 3, some denuded tips of the villi and massive lifting of the epithelial layer; Grade 4, dilated and exposed capillaries and denuded villi; and Grade 5, haemorrhage, ulceration and disintegrated lamina propria [19]. Histologic analyses were performed by two blinded observers.

The left lung was cut into sections and fixed in 40 g/L formaldehyde. After being embedded in paraffin, 4 μm sections were stained with haematoxylin and eosin for light microscopy. Damage to the lung tissue was graded by the pathologist on a scale of 0 (best) to 4 (worst), according to combined assessments of alveolar congestion, hemorrhage and edema, infiltration/aggregation of neutrophils in the airspace or vessel wall, thickness of the alveolar wall, and hyaline membrane formation. Lung injury score was calculated as the mean of the scores for the separate parameters [20]. Histologic analyses were performed by two blinded observers.

### 3.4. Intestinal and Lung MDA Assay

It is well documented that lipid peroxidation due to I/R is one of the main causes for lung injury [21]. Malondialdehyde (MDA) levels in tissue samples were determined as an indicator of lipid peroxidation. The intestinal and lung tissues were harvested and immediately homogenized on ice in 5 volumes of normal saline. The homogenates were centrifuged at 1,200 g for 10 min. The MDA levels in the supernatant were measured using an MDA assay kit (Nanjing Jiancheng Bioengineering Institute, Nanjing, China) according to the manufacturer’s instructions.

### 3.5. Lung Wet Weight to Dry Weight Ratio

To quantify the magnitude of pulmonary edema, we evaluated lung water content. The right lung was excised, weighed, and then dried at 73 °C for 72 h. The residuum was weighed, and the ratio of wet weight to dry weight was estimated. 

### 3.6. Lung MPO and TNF-α Assay

Myeloperoxidase (MPO) activity was detected to assess neutrophil sequestration. Proinflammatory cytokines are major contributors in the injury of remote organs after intestinal I/R. The lung tissues were harvested and immediately homogenized on ice in five volumes of normal saline. The homogenates were centrifuged at 1,200 *g* for 10 min. The MPO and TNF-α levels in the supernatant were measured using assay kits according to the manufacturer’s instructions. One unit of MPO activity was defined as degrading 1 mmol hydrogen peroxide/g tissue at 37 °C and expressed as U/g.

### 3.7. Lung ICAM-1 Immunohistochemical Assays

Paraffin-embedded lung sections were stained using the streptavidin peroxidase (SP) immunohistochemistry technique for ICAM-1 detection. After being dewaxed or washed in phosphate-buffered saline (PBS), 4 μm sections were immersed in 3% hydrogen peroxide to eliminate intrinsic peroxidase, quenched in normal goat serum for 30 min, and the sections incubated overnight at 4 °C with polyclonal rabbit anti-rat ICAM-1 antibody (1:200 dilutions), against purified recombinant ICAM-1. Then, anti-rabbit immunoglobulin and streptavidin conjugated to horseradish peroxidase were added. Finally, 3,3'-diaminobenzidine (DAB) was used for colour development and haematoxylin was used for counterstaining. Brown staining in the cytoplasm was considered an indicator of positive expression. Results were evaluated semiquantitatively according to optical density values of positive expression with the Medical Image Analysis System HMIAS-2000 (Qianping Image Co., Wuhan, China). 

### 3.8. Lung NF-kB Western Blot Analysis

Cellular nuclear proteins were extracted from frozen lung tissue with a nuclear extract kit according to the manufacturer’s instructions. An equal amount of protein was loaded onto 10% sodium dodecyl sulphate polyacrylamide gel electrophoresis (SDS-PAGE) at 100 V for 3 h. After electrophoresis, proteins were transferred onto nitrocellulose membranes at 200 mA for 2 h. The transferred membranes were incubated overnight at 4 °C with mouse polyclonal antibodies NF-kB p65 (1:1000 dilution) against rat in PBS-T containing 5% skim milk. After washing three times in Tris phosphate-buffered sodium (TBS-T), membranes were incubated with anti-mouse IgG conjugated to horseradish peroxidase at a dilution of 1:2,000 in PBS-T containing 5% skim milk for 1h at room temperature. The immunoreactive bands were visualized with enhanced chemiluminescence (ECL) and captured on X-ray film. Optical density of the bands was measured with a Gel imaging analysis system. 

### 3.9. Statistical Analysis

Mean ± SD values were calculated to summarize all outcome measurements. One-way analysis of variance (ANOVA) was used to compare the means of four groups and *p* < 0.05 was chosen to indicate statistical significance.

## 4. Conclusions

Our results indicated that Rb1 markedly attenuated lung injury by inhibiting NF-kB activation in lung tissue. The inhibition of NF-kB pathway may be involved in the protective effects. 
